# A close call: does the location of incision at cesarean delivery matter in patients with vasa previa? A case report.

**DOI:** 10.12688/f1000research.2-267.v1

**Published:** 2013-12-05

**Authors:** Werner M Neuhausser, Laxmi V Baxi

**Affiliations:** 1 Division of Reproductive Endocrinology & Infertility, Beth Israel Deaconess Medical Center/Boston IVF, Harvard Medical School, Boston, MA, USA; 2 Adjunct Professor Ob-Gyn, New York University School of Medicine, New York, NY, USA; 3 Professor Emerita, Clinical Obstetrics and Gynecology, Columbia University, New York, NY, USA

## Abstract

We present here a case of vasa previa in a multipara, diagnosed at the time of her late second trimester ultrasonogram. The patient subsequently underwent an elective cesarean section after 37 weeks gestation, giving birth to a healthy child with an uneventful post-partum, neonatal and infant course. At the time of cesarean section, the incision was gradually deepened in layers through the myometrium by utmost care allowing the amniotic sac to protrude through the uterine incision hereby avoiding laceration of the vasa previa and its branches. Fetal exsanguination and a need for blood transfusion as well as a possible adverse neonatal course were therefore avoided.

## Case

The CARE checklist submitted with this case report is accessible here.

**Figure 1.**
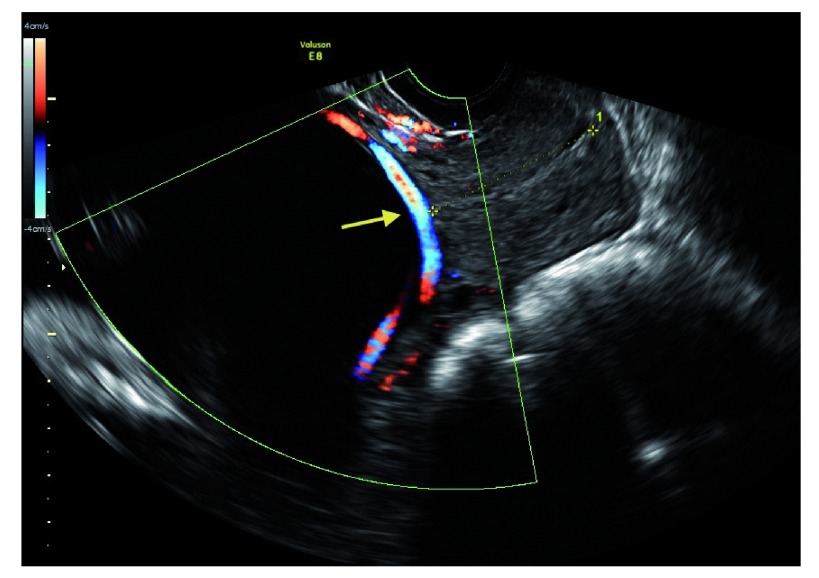
Doppler images showing blood flow through a vasa previa vessel over the internal os between the anterior placental cord insertion and the posterior succenturiate lobe (arrow).

A 27 year old, Caucasian gravida 4 para 3, was diagnosed with vasa previa at a 22 week anatomy scan. On ultrasound her right lateral placenta had a significant anterior component with a marginal cord insertion at the inferior margin of its anterior aspect. A vessel coursing over the internal os between the anterior placental cord insertion and a posterior succenturiate lobe was identified on ultrasound (type 2 vasa previa, see Figure 1). The patient’s antenatal course was otherwise uneventful. She was counseled for Cesarean delivery at 35–36 weeks to avoid the risk of inadvertent rupture of the vasa previa and fetal exsanguination ^1, 2^. However, she believed that considering given her past obstetrical history of post –term pregnancies and her closed long cervix, she is was less likely to go into preterm labor or sustain preterm premature rupture of membranes with a disastrous outcome secondary to vasa previa. She therefore declined hospitalization and requested delivery after 37 weeks gestation.

## Treatment

**Figure 2.**
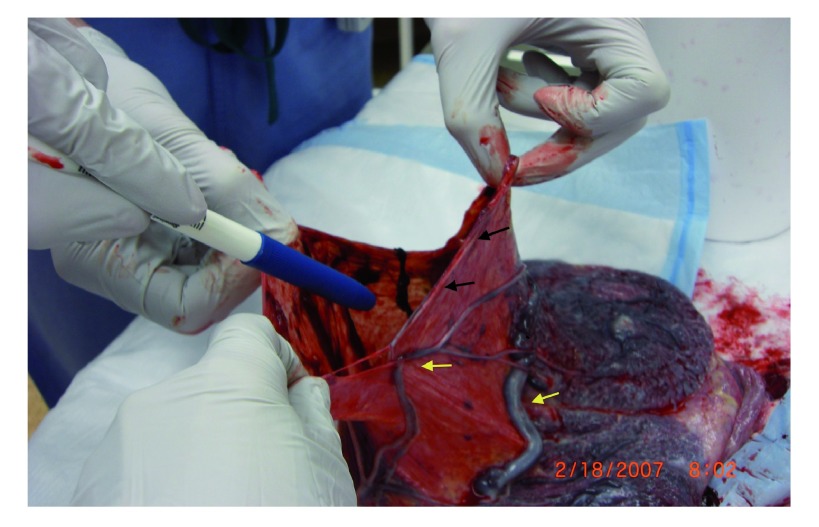
Amniotic sac showing presence of vasa previa vessels running in juxtaposition to the incision through which the baby was delivered, thus avoiding severance of the vessels. The site of amnion incision (black arrows) in the vicinity of but distinct from the vasa previa vessels (yellow arrows).

Primary low transverse Cesarean section was performed at 37 weeks and 1 day gestation as per the patient’s request. At the time of surgery, the uterine incision was gradually and carefully deepened to allow the membranes to remain intact and bulge out from the incision. This was to avoid making an incision into the amniotic sac prior to localization of the course of the vasa previa vessels in the exposed membranes underneath the uterine incision. Indeed, extensive vasa previa vessels were identified in the intact membranes directly underneath the uterine incision in the lower uterine segment ( Figure 2). After identification of the vasa previa vasculature the amnion was incised about one cm away, remaining parallel to the vessel leaving these vessels intact. Additionally, hemostats were kept available to clamp the vessel on either side if possible extension of the incision into one of the vessels occurred; and followed by expeditious delivery of the baby. A healthy 2980 g male fetus was delivered with Apgar scores 9, 9 and umbilical cord artery (Ua) pH 7.3.

## Discussion

Vasa previa is a rare (1:2500) but important and potentially fatal cause of bleeding in the second and third trimester as well as in labor. The condition carries a risk of fetal exsanguination and death when rupture of the membranes involves tearing of vasa previa vessels running within the membranes and carrying fetal blood. Fortunately, the condition can often be diagnosed prenatally by ultrasound examination. Type I vasa previa refers to velamentous insertion of the cord with resultant vasa previa and Type II indicates interconnecting vessels between two lobes of placenta in a bipartite placenta or connecting vessel with a succenturiate lobe of the placenta. Nomiyama *et al.* identified placental cord insertion site with great degree of certainty at 18–20 weeks gestation and Sepluveda confirmed that gray scale with color Doppler has significant and better accuracy in diagnosing potential abnormal cord insertion and exclude vasa previa than 3D ^3, 4^. Situations, where vasa previa should be specifically looked for, include a larger placental mass as it is seen in multiple gestations, particularly those with a high number of fetuses, where there is a greater likelihood of velamentous insertion of the cord, succenturiate lobe or bipartite placenta. Patients with a low lying placenta, particularly if the placental margin appears at the internal os, and pregnancies conceived following *in-vitro*-fertilization constitute ‘at risk’ groups as well. Favorable outcomes depend on prenatal diagnosis and Cesarean delivery before the rupture of membranes. However, transection of vasa previa vessels during Cesarean delivery itself may cause significant fetal blood loss or exsanguination given the small fetal blood volume. Although the chance of fetal exsanguination during Cesarean section is less common than during vaginal delivery this complication, requiring blood transfusion to the newborn, has been reported ^4^. This can be avoided by careful selection of the site of amniotic incision prior to delivery of the fetus. Canterino *et al.* recommend use of 3D sonography with power Doppler imaging with surface rendering and 3D multiplannar reconstruction to confirm vasa previa and also to map the path of this fetal vessel to prevent laceration of the vessel at cesarean section and fetal exsanguination ^5^. According to Oylese *et al.*, although 2D and color Doppler sonography is mostly adequate, 3D “allowed precise depiction of complex spatial relationship and confirmed vasa previa” and has an important role in placental abnormalities with uncertain diagnosis ^6^. Vaginal ultrasonography (USG) with color Doppler is a recommended way to identify or confirm vasa previa ^7^. In a recent case report, the authors published fetoscopic laser coagulation of a type II vasa previa at 32 5/7 weeks gestation and thereby facilitated an uneventful vaginal delivery, though the authors rightly caution benefits vs. risk of fetoscopy ^8^. Earlier, Quintero *et al.* and others have published early third trimester *in-utero* laser treatment of type II vasa previa, however these patients had preterm delivery and needed cesarean section ^9, 10^. Although pre-op mapping of vasa previa by USG is an excellent measure to identify the course of the vasa previa vasculature, considering the recommendations made in this case report could be of additional help in the management of these patients.

## Patient perspective

I would like to thank Dr. Baxi for the most professional, delicate care she provided during my pre-natal care and through the high risk cesarean section.

## Consent

The patient has given written consent for publication of these findings and believes sharing of this information would benefit other patients.
