# Supplementary material for: A close call: does the location of incision at cesarean delivery matter in patients with vasa previa? A case report
Source: F1000Res. 2013 Dec 5;2:267. [Version 1] doi: 10.12688/f1000research.2-267.v1 (PMC4103493; doi:10.12688/f1000research.2-267.v1)
Supplement: Peer review history

# Peer review: Referee response for version 1

**DOI:** 10.5256/f1000research.3140.r4572

**Published:** 2014-07-10

**Authors:** Ryu Matsuoka

**Affiliations:** 1 Department of Obstetrics and Gynecology, Showa University, Tokyo, Japan

This is the case report of vasa previa. It was well described in detail and very helpful to the physician.

I have read this submission. I believe that I have an appropriate level of expertise to confirm that it is of an acceptable scientific standard.

# Peer review: Referee response for version 1

**DOI:** 10.5256/f1000research.3140.r5127

**Published:** 2014-06-16

**Authors:** Everett Magann

**Affiliations:** 1 Department of Obstetrics, University of Arkansas for Medical Sciences, Little Rock, AR, USA

This case report is well written, insightful, and addresses the problems linked with the cesarean delivery for a vasa previa. It describes a technique to ensure the integrity of the fetal vessels located within the membranes as a cesarean delivery is undertaken.

I have read this submission. I believe that I have an appropriate level of expertise to confirm that it is of an acceptable scientific standard.

# Peer review: Referee response for version 1

**DOI:** 10.5256/f1000research.3140.r2724

**Published:** 2014-01-30

**Authors:** Conrad Chao

**Affiliations:** 1 University of California, San Francisco, San Francisco, CA, USA

This is an interesting case report with useful suggestions for clinical practice.

I have read this submission. I believe that I have an appropriate level of expertise to confirm that it is of an acceptable scientific standard.
